# Dysregulation of protein synthesis and dendritic spine morphogenesis in ASD: studies in human pluripotent stem cells

**DOI:** 10.1186/s13229-020-00349-y

**Published:** 2020-05-27

**Authors:** Louisa Hoi-Ying Lo, Kwok-On Lai

**Affiliations:** 1 School of Biomedical Sciences, Faculty of Medicine, The University of Hong Kong, 21 Sassoon Road, Pokfulam, Hong Kong, China; 2 State Key Laboratory of Brain and Cognitive Sciences, The University of Hong Kong, Pokfulam, Hong Kong, China

## Abstract

Autism spectrum disorder (ASD) is a brain disorder that involves changes in neuronal connections. Abnormal morphology of dendritic spines on postsynaptic neurons has been observed in ASD patients and transgenic mice that model different monogenetic causes of ASD. A number of ASD-associated genetic variants are known to disrupt dendritic local protein synthesis, which is essential for spine morphogenesis, synaptic transmission, and plasticity. Most of our understanding on the molecular mechanism underlying ASD depends on studies using rodents. However, recent advance in human pluripotent stem cells and their neural differentiation provides a powerful alternative tool to understand the cellular aspects of human neurological disorders. In this review, we summarize recent progress on studying mRNA targeting and local protein synthesis in stem cell-derived neurons, and discuss how perturbation of these processes may impact synapse development and functions that are relevant to cognitive deficits in ASD.

## Background

Autism spectrum disorder (ASD) is a neurodevelopmental disorder characterized by social interaction failure, anxiety, intellectual disability, and repetitive behavior. According to the statistics from the Centers for Disease Control and Prevention, 1 in 59 children is diagnosed with ASD which affects all ethnic and socioeconomic groups [1]. ASD is 4 times more common among boys than girls and can be diagnosed as early as age of 2. The exact cause of ASD is still unclear, but the occurrence of ASD is highly associated with both genetic and environmental risk factors. Fragile-X syndrome (FXS), tuberous sclerosis complex (TSC), and Rett syndrome are among some of the common syndromic ASD which are caused by monogenetic defects, and the corresponding genes that cause FXS (*FMR1*), TSC (*TSC1* and *TSC2*), and Rett syndrome (*MECP2*) have been identified [2]. However, it is now clear that genetic variants of an extensive network of genes are involved as contributing factors for ASD, and more than 1000 human ASD-risk genes have been reported in the Simons Foundation Autism Research Initiative (SFARI) database. Notably, many ASD-risk genes seem to converge onto common signaling pathways that perturb specific neuronal functions [3, 4]. One major cellular process that is disrupted in ASD is the development of excitatory synapses.

### Dendritic spines and autism spectrum disorder

Most excitatory synapses are located on dendritic spines of the postsynaptic neuron. These small (typically < 1 μm) yet dynamic dendritic protrusions exist as various morphologies [5]. These include the mushroom spines containing large “mushroom-shaped” heads that are separated from the dendrite by well-defined spine necks, the stubby spines which are short and lack of spine neck, and the thin spines which have long spine necks and small bulbous heads. There is a fourth type of dendritic protrusions called filopodia which are more abundant during early stage of neuronal development. Filopodia are long and thin without distinct spine head, and they are believed to actively search for presynaptic partners to initiate the formation of neuronal connections during synaptogenesis [6].

Spine morphology is a crucial determinant of structural stability and functions of the synapse. Mushroom spines have better synaptic efficacy than thin spines because of the positive correlation between spine size and synaptic strength [7, 8], in which the number of postsynaptic receptors and the size of the postsynaptic density (PSD) depend on the spine head volume [9]. As neurons mature, the mushroom spines become more abundant and replace the immature filopodia [10]. This transition of dendritic spine morphology is required for higher cognitive functions such as learning and memory formation. On the other hand, the width of the spine neck is crucial for compartmentalization of synaptic signals [11], while the length of dendritic spine can alter many physiological properties such as synaptic membrane tension which regulates the insertion of glutamate receptors [12]. The tight regulation of dendritic spine morphology is therefore crucial for synaptic function and plasticity.

Alteration of spine number and morphology is believed to underlie many neurological disorders including ASD [13]. Analysis of postmortem brain samples indicates higher dendritic spine densities in cortical neurons from ASD patients [14]. Dendritic spine defects are also associated with other autism-related monogenetic disorders. In FXS, high portion of immature filopodia is observed as compared to control individuals [15]. Two-photon imaging of cortical neurons in FXS mouse model further shows delay in spine maturation and downregulation of spine turnover during the first two postnatal weeks [16]. In Rett syndrome, the spine density is reduced and is coupled with lower proportion of mushroom spines. For TSC, there are fewer spines during spinogenesis, but higher spine density is observed during spine maintenance [17]. Cultured neurons that model TSC display early defects during spinogenesis which is characterized by overabundance of filopodia [18, 19], while spine pruning is impaired which leads to higher spine densities [20].

Dendritic spines are rich in filamentous actin. Spine morphology and number are intricately controlled by signaling pathways that involve multiple small GTPases (such as those belong to the Rho, Ras, and Rap families), which act on various actin-binding proteins to modulate actin dynamics. The activities of the small GTPases in turn depend on guanine nucleotide exchange factors (GEFs) and GTPase-activating proteins (GAPs), which regulate their GTP loading state and hence activation of the downstream targets. Dysfunction of GEFs and GAPs that act upstream of Ras and Rap is associated with ASD. For example, truncated form of SynGAP, which is a major neuronal GAP that controls the activity of Ras, is expressed in individuals with ASD and intellectual impairment [21, 22]. On the other hand, the missense mutation on the Rap-GEF Epac2 resulting in the G706R substitution is detected in individuals with ASD [23]. Other strong ASD candidate genes that control the actin cytoskeleton include *CYFIP1* and *CTTNBP2*. Therefore, alteration in actin dynamics and the subsequent dendritic spine defects are one of the major underlying causes for ASD and other ASD-related disorders.

### Local protein synthesis and dendritic spine morphogenesis

The discovery that polyribosomes are dendritically localized in the granule cells of the rat dentate gyrus [24] raises the intriguing possibility that protein synthesis can occur on dendrites. This is further supported by the observation that synapse-enriched fractions containing dendritic fragments without the cell body are able to synthesize proteins [25]. Protein synthesis taken place on dendrites allows higher degree of flexibility towards local changes in synaptic composition and responses to neuronal activity, which helps to shape brain connections according to need [26]. Local protein synthesis near synapses is possible only if mRNAs can be transported from soma to distal dendrites. Dendritic mRNA transport is first validated by the detection of mRNAs encoding microtubule-associated protein 2 (MAP2) in the dendrites of cultured hippocampal neurons [27]. The transport of mRNA transcripts depends on specific RNA-binding proteins (RBPs), which form ribonucleoprotein complexes with the dendritic mRNAs and transported to the distal dendrites via the action of motor proteins such as kinesin and dynein [28–32]. RBPs provide tight regulation in dendritic mRNA transport by repressing the translation during trafficking and release the transcripts at the endpoint for translation in response to neuronal activity [33].

Local dendritic protein synthesis is crucial for controlling spine size and morphology. The increasing spine head volume in CA1 pyramidal neurons of hippocampal slices during to synaptic plasticity such as long-term potentiation (LTP) is attenuated by the application of protein synthesis inhibitors [34]. Messaoudi et al. further showed that the early synthesis of Arc is required for the expression of LTP. Knockdown of Arc leads to dephosphorylation of cofilin and loss of nascent F-actin at synaptic sites [35]. This study suggests a model in which dendritic synthesis of Arc promotes filamentous-actin expansion and triggers the structural changes of synapses that underlie stable LTP. It is generally believed that the cis-acting elements located in the 3′-untranslated region (3′-UTR) are responsible for dendritic localization of mRNAs. These include the *Bdnf* and *CaMkIIα* transcripts, two of the best characterized dendritic mRNAs [36–38]. Notably, transgenic mice with disrupted 3′-UTRs of those transcripts but normal coding regions exhibit abnormal spine maturation, synaptic dysfunction, and impaired spatial memory [39, 40]. Furthermore, the Val66Met polymorphism of BDNF, which is associated with anxiety and depression [41], also impairs the dendritic targeting of *Bdnf* transcripts [42]. It therefore appears that local protein synthesis is essential and cannot be compensated by delivery of protein products derived from somatic mRNA translation, and this compartment-specific synthesis of new proteins regulates dendritic spine development for proper cognitive functions.

The identities of dendritic mRNAs were elucidated by multiple large-scale transcriptomic studies. Deep sequencing analysis by Cajigas et al. revealed the local transcriptome of the synaptic neuropil in adult hippocampus. More than two thousand mRNA transcripts have been identified, most of which encoded synaptic proteins such as receptors, scaffold proteins, and signaling proteins [43]. Other high-throughput RNA sequencing studies also revealed nearly 2000 dendritically localized mRNAs [44, 45]. Through characterizing the proteins encoded by dendritically localized transcripts, novel regulatory pathways that control postsynaptic development may be identified, which potentially provide new insights into the pathophysiology of brain disorder [46–48]. With regard to ASD, it is noteworthy that ASD-risk transcripts such as *Shank* and *Cyfip* have been identified as transcripts in the neuropil in those high-throughput studies. Shank is a scaffold protein present in the excitatory PSD. In human, the proteins are encoded by *SHANK* genes (*SHANK1*, *SHANK2*, and *SHANK3*) and their mutations are strongly associated to ASD [49]. Overexpression of *Shank3* mutants (R12C and R300C) in cultured hippocampal neurons causes Shank3 dysfunction and disrupts spine induction and maturation [50]. CYFIP1 (cytoplasmic FMRP-interacting protein 1) is a binding partner of FMRP, and it represses mRNA translation through binding to the translational initiation factor eIF4E [51]. Interestingly, CYFIP1 is also part of the WAVE complex that promotes actin polymerization by interacting with the Arp2/3 complex, thereby likely contributing to the abnormal spine morphogenesis in FXS. Copy number variations on *CYFIP1* have recently been associated with ASD and are believed to alter the balance between synaptic excitation and inhibition [52].

### Aberrant protein synthesis and mRNA processing in ASD

Dysregulated protein synthesis is a plausible mechanism underlying the synaptic deficits in ASD [53, 54], and correction of protein synthesis has been implicated as a potential therapeutic approach [55, 56]. FXS is caused by loss of the RBP Fragile-X mental retardation protein (FMRP), which is resulted from expansion of CGG repeats in the promotor of the *FMR1* gene, leading to hypermethylation and silencing of transcription. The loss of FMRP production affects dendritic mRNA transport and translational regulation as well as dendritic spine maturation [57]. For example, in FMRP knockout neurons, there is increased expression of the bone morphogenetic protein type II receptor (BMPR2) which activates the kinase LIMK1 to increase phosphorylation and inhibition of the actin-depolymerization factor cofilin, thereby altering actin dynamics and spine growth [58]. Many FMRP targets are mRNAs that are encoded by ASD candidate genes listed in the SFARI database [59, 60], underscoring the important link between FMRP-mediated control of local protein synthesis and ASD.

Disrupted control of mRNA translation also causes spine defects and autistic behaviors [53, 56, 61, 62]. TSC, another syndromic form of ASD, is caused by the mutation of *TSC1* or *TSC2* genes which fail to control the activity of mammalian target of rapamycin (mTOR). Alteration in mTOR pathway leads to exaggerated protein translation in dendrites and resulting in intellectual disability [63, 64]. Interestingly, a recent study shows that splicing of transcripts encoding the translational regulator CPEB4, which is present at postsynaptic sites to promote translation of dendritic mRNAs through polyA elongation, is affected in idiopathic ASD and leads to reduced expression of many ASD-risk genes [65]. Therefore, in addition to syndromic ASD such as FXS and TSC, local dendritic mRNA translation may also be disrupted in the more common idiopathic ASD.

Emerging studies reveal that besides translation, other aspects of post-transcriptional regulation of RNA processing such as RNA splicing and editing are also dysregulated in ASD [66–68]. Given that alternative splicing which generates distinct 3′-UTRs and RNA editing can both affect dendritic mRNA localization [69, 70], it is plausible that aberrant RNA splicing and editing observed in ASD will lead to alteration in the dendritic transcriptome and subsequently affecting local protein synthesis.

### Dendritic mRNA and protein synthesis in pluripotent stem cell-derived neuron

Most of our knowledge on the physiology of neurodevelopmental disorders such as ASD depends on rodent animal models. While rodent animal models can be conveniently used to screen molecular targets for various neurological disorders, it has limitations in mimicking the complex genetic background in human. Examination of postmortem human brain samples offers some insights on the abnormal neuronal properties such as dendritic spine number and morphology in neurons of ASD patients, but postmortem samples do not allow mechanistic studies which involve manipulation of living neurons. The development of induced pluripotent stem cell (iPSC) technology was introduced in 2006 which allows the conversion of adult somatic cells into pluripotent stem cells [71]. Recent literature has discussed the protocols for effective generation of iPSC-derived neural stem cells and neuronal differentiation [72]. The study of iPSC-derived neurons from somatic cells of healthy individuals and diseased subjects can help to broaden our understanding on human physiology and disease therapeutics. Apart from iPSCs, human embryonic stem cells (hESC) have been frequently used for differentiation into human neurons. Despite the blossoming of studies in recent years on iPSC- or ESC- (collectively referred to as pluripotent stem cells or PSCs) derived neurons and their use in modeling neurodevelopmental diseases, there are relatively few studies that focus on mRNA targeting and regulation of protein synthesis in stem cell-derived neurons. Given the strong association between ASD and dysregulation of mRNA translation discussed above, it is important to understand whether mRNAs are similarly transported to neurites of stem cell-derived neurons as in rodent primary neurons, followed by characterization of local translation in iPSCs from ASD patients. In this section, we will summarize the recent findings reporting the axonal and dendritic mRNA localization as well as local protein translation in stem cell-derived neurons.

Using the compartmentalized microfluidic chambers to evaluate the axonal transcriptome of hESC-derived glutamatergic neurons, large number of mRNAs (3696 transcripts) are found to be expressed in the distal axonal projections of hESC-neurons, and this axonal transcriptome is largely similar to the primary rat cortical neurons counterparts [73]. However, this study has also identified transcripts, such as those encoding oxytocin, that may be uniquely localized to axons of human but not mouse neurons, indicating that human neurons might possess mechanisms of mRNA targeting distinct from rodent. Further evidence for mRNA targeting and local translation has been gained in a recent high-throughput study of mouse ESC-derived neurons, which were obtained through exogenous expression of the pro-neuronal factor ASCL1. The neurons were cultured on microporous membrane in order to separate the neuritic compartments from the soma [74]. Through analysis of the two fractions by mass spectrometry, RNAseq and ribosome profiling, the authors show that about half of the proteins enriched in the axons and dendrites of ESC-derived neurons originate from locally translated mRNAs. Puromycin-proximity ligation assay was further performed to demonstrate local translation of selected transcripts in the dendrites. This study therefore conclusively shows that local dendritic translation is a prominent process in stem cell-derived neurons that contributes substantially to the neuronal polarity and asymmetric distribution of proteins. It would be important for similar high-throughput study to be performed in human stem cell-derived neurons and determine if the neuritic transcriptome is similar across the two species.

Does dendritic local translation affect synapse functions in human iPSC-derived neuron? Olfactory placodal neurons differentiated from patient-derived iPSCs carrying microdeletions of the *SHANK3* gene show abnormal morphology, including smaller soma and more neurites, prior to synaptogenesis. This is followed by decrease in pre- and postsynaptic puncta number in the more mature neurons, indicating the crucial role of SHANK3 in neuronal development and synapse formation [75]. iPSC-derived cortical neurons isolated from four ASD patients harboring the autism risk gene *SHANK3* de novo heterozygous truncating mutations also display significant reduction in *SHANK3* mRNA levels and a decrease in dendritic spine density and spine head volumes [76]. Using single-molecule fluorescence in situ hybridization (smFISH), it was found that *SHANK3* mRNA puncta were visible in both cell soma and neuronal processes of human iPSC-derived cortical neurons, while SHANK3 protein expression is only dendritically localized [77]. This is consistent with previous findings of the direct visualization of *Shank* dendritic transcripts from rodent neurons [78], in which the 3′-UTR of *Shank1* transcripts contains a dendritic targeting element that mediates dendritic localization. Interestingly, cortical neurons differentiated from iPSC of ASD patients with *SHANK3* heterozygous deletion showed ~ 50% reduction in *SHANK3* mRNA only within neuronal processes but not in the cell soma [77]. Therefore, key genes that control synapse development such as *SHANK3* are likely to undergo local translation in neuronal dendrites, and deficiency of the local translation alone is sufficient to lead to synaptic changes in ASD.

One important question that has not been well-addressed is whether aberrant protein synthesis is observed in human stem cell-derived neurons modeling ASD. While study that specifically examines dendritic local protein synthesis is not yet available, global protein synthesis as measured by metabolic labeling with radioactive-labeled Cys/Met is downregulated in human ESC-derived neurons that model Rett syndrome through knockout of the *MECP2* allele [79]. This is surprising given that the lack of MECP2 in Rett syndrome is expected to disrupt transcription rather than translation, and the result is possibly explained by the reduced transcription of genes encoding ribosomal proteins as well as BDNF, one of the major growth factors that induces neuronal protein synthesis through the Akt/mTOR pathway. Notably, knockdown of PTEN, a negative regulator of PI3K acting upstream of the Akt/mTOR signaling, can reverse the defective neuronal morphology in the *MECP2* knockout ESC-derived neurons, indicating that correction of aberrant protein synthesis is a promising therapeutic strategy for ASD. While Rett syndrome neurons may represent one end of the ASD spectrum showing reduced protein synthesis, it is anticipated that human PSC-derived neurons that model ASD from FXS, TSC, or eIF4E mutations will display elevated protein synthesis. Elegant approaches involving super-resolution live imaging have been developed to monitor the synthesis of nascent polypeptides in rodent primary neurons [80]. Examining protein synthesis in real time and elucidating the spatial and temporal changes in protein synthesis in stem cell-derived neurons that model ASD should represent an exciting research area to pursue in the near future.

### Synaptic defects of human PSC-derived neurons carrying mutations that affect mRNA translation

Although direct examination on the effect of ASD mutations on dendritic mRNA localization or protein synthesis in human stem cell-derived neurons is lacking, synaptic deficits have been observed in human stem cell-derived neurons harboring ASD-related gene mutations that are expected to perturb mRNA targeting and/or translation. TSC is a disorder that is associated with syndromic form of ASD. In *TSC2*-deleted human iPSC-derived neurons, hyperactive mTOR signaling leads to reduced excitability and excitatory postsynaptic currents (EPSCs), and the defects are corrected by the mTOR inhibitor rapamycin [81]. Similarly, cerebellar Purkinje cells differentiated from TSC patient-derived iPSCs also displayed hypoexcitability, decreased expression of synaptic markers, and miniature EPSCs [82]. Interestingly, these patient-derived iPSCs with heterozygous *TSC2* mutation also show reduced expression of transcripts encoding dendritic RNA-binding proteins including FMRP, suggesting that the absence of TSC2 can disrupt dendritic mRNA transport and protein synthesis which may subsequently contribute to the synaptic deficits. Recent study also reports differential effects of single or biallelic mutation on the *TSC2* gene on patient-derived iPSC neurons, with increased synchrony of neuronal activity only observed in TSC^−/−^ neurons [83].

FMRP is crucial for dendritic protein synthesis by transporting the mRNA cargoes and regulating their translation [84]. Multiple studies have characterized FXS patient-derived stem cells and the differentiated neurons. Neurite outgrowth during early differentiation is impaired in iPSC-derived neurons originated from FXS patients [85, 86], indicating that FMRP is crucial for the initial early development and making it difficult to conclude the specific role of FMRP in synapse development. On the other hand, neurons differentiated from ESCs of FXS blastocysts have normal neurite outgrowth, but they are less mature than the wild-type counterparts by displaying abnormality in action potential and reduction of spontaneous synaptic activity which is associated with decrease in presynaptic functions [87]. Interestingly, by comparing iPSC-derived neurons from a female premutation carrier which contains mixed populations of cells with different lengths of the CCG repeats, it was found that the presence of extended CCG repeats in the *FMR1* gene promotor without change in FMRP protein expression can negatively affect neurite growth and synapse development [88], indicating gain-of-toxicity effect of *FMR1* mRNA on neuronal function. Furthermore, FMRP plays multi-faceted roles in neuron. Besides mRNA transport and translational regulation, FMRP is also crucial for epigenetic control, chromatin stability, and DNA damage response [84]. Therefore, the causative link between synaptic defects in FXS patient-derived neurons and protein synthesis should be interpreted with care, as the phenotype may be caused by dysregulation of other cellular processes besides local protein synthesis.

Although overproduction of immature spines has been one of the major phenotypes in neurons lacking FMRP in both knockout mice and FXS patients [15], thus far the studies on iPSC- or ESC-derived neurons from FXS patients have not examined the number and morphology of dendritic spines. However, insights on the potential role of FMRP signaling in spine morphogenesis of human neuron may be indirectly provided through the study on CYFIP1, which interacts with FMRP and mediates the translation repression by FMRP as well as regulating actin dynamics through Rac1 and the WASP family member (WAVE) complex [89]. Disruption of CYFIP expression is associated with intellectual disability and ASD. Human iPSCs and iPSC-derived neurons with autism-risk copy number variation on chromosome 15q11.2 show reduction in *CYFIP1* gene expression [90], and these neurons display 7.5-fold increase in filopodia density compared to neurons from control individual [91]. It should be noted that the micro-deletion on chromosome does not only affect the *CYFIP1* gene but also the neighboring genes *NIPA1*, *NIPA2*, and *TUBGCP5*, which encode proteins for Mg^2+^ transport and tubulin-interacting protein, respectively. Future work on human iPSCs with *CYFIP1* deletion generated by genome editing should validate the specific role of this protein in the control of actin dynamics and spine maturation.

### Challenges and promises of iPSC-derived neurons in the study of protein synthesis and dendritic spine morphogenesis

In this review, we have summarized the recent attempts to examine mRNA targeting, protein synthesis, synaptic transmission, and dendritic spine morphogenesis using human pluripotent stem cell-derived neurons that should have important implications in future study of ASD. There are potential advantages and pitfalls in the study of local protein synthesis and dendritic spine morphology defects in human pluripotent stem cell-derived neurons. On one hand, the culture of patient-derived iPSCs can be scaled up to provide enough quantity of RNAs and proteins that are essential for high-throughput transcriptomic and proteomic studies. Ribosome profiling and RNAseq of neurites versus cell soma [74] in human stem cell-derived neurons from patients with monogenetic ASD-related disorders such as FXS and TSC as well as idiopathic ASD will be crucial to address how local protein synthesis is disrupted in ASD. These neurons can also be used to test the validity of correcting protein synthesis as a feasible strategy to reverse defects in neuronal properties such as synaptic transmission and dendritic spine morphology. On the other hand, the number of patient lines studied in iPSC studies are not large and may not be sufficient to validate the hypothesis linking protein synthesis with dendritic spine morphogenesis. Towards this end, it would be desirable to confirm findings of monogenetic ASD patient-derived iPSC by generating isogenic iPSCs that harbor the particular gene mutations, which can now be readily achieved by genome editing such as CRISPR-Cas9. Furthermore, the maturity of dendritic spines formed in cultured human stem cell-derived neurons may also be a concern for detailed study of dendritic spine morphology defects in ASD [90, 91], although the presence of mature spines in iPSC-derived neurons has been recently demonstrated [76]. Synapse maturation may be enhanced by co-culture between human iPSC-derived neurons and mouse hippocampal neurons in slices, in which the human neurons can be integrated into the preexisting neural circuits in a region-specific manner. This co-culture allows efficient and specific differentiation of transplanted iPSC-derived neurons and the formation of more mushroom spines [92]. Moreover, organoids which are self-organized three-dimensional tissue derived from stem cells are well-suited to mimic the complexity of human organs. With the technological advancement of 3D printing, miniaturized spinning bioreactor has been developed to generate forebrain, midbrain, and hypothalamus organoids from human iPSCs [93]. The development of human brain organoids that are maintained for extended period (more than 9 months) allows the development of mature dendritic spines with presynaptic contacts [94]. Synaptosome can be isolated from human brain organoid [95], which can be a useful source for future identification of synaptic mRNAs that undergo local translation in hiPSC-derived neurons [96, 97]. The advancement of organoids would promise to be an exciting tool for exploring the link between postsynaptic developmental defects and dysregulated local protein synthesis in human ASD neurons.

## Conclusion

ASD is strongly associated with aberrant protein synthesis which can lead to impairment of dendritic spine development. Application of human iPSCs has greatly enhanced the generation of human cellular models to unravel the causes and mechanisms that underlie inherited neurological disorders like ASD. Synaptic deficits are observed in ASD patient-derived iPSC neurons that carry mutations to disrupt mRNA translation, supporting the causal link between uncontrolled protein synthesis and ASD. Nonetheless, there are limited studies on dendritic mRNA targeting and protein synthesis in human pluripotent stem cell-derived neurons. In addition, technical difficulties of obtaining those neurons that possess mature dendritic spines remain. It is anticipated that the rapid advance of imaging, transcriptomic analysis, and development of brain organoids will soon facilitate the exploration on dendritic protein synthesis in human neurons, which allow us to understand how its regulation is altered in different ASD patients.

## Data Availability

Not applicable. There is no data in this review article to be available.

## Abbreviations

3′-UTR: 3′-untranslated region
ASD: Autism spectrum disorder
BMPR2: Bone morphogenetic protein type II receptor
CYFIP1: Cytoplasmic FMRP-interacting protein 1
FMRP: Fragile-X mental retardation protein
FXS: Fragile-X syndrome
GEF: Guanine nucleotide exchange factor
GAP: GTPase-activating protein
hESC: Human embryonic stem cell
iPSC: Induced pluripotent stem cell
LTP: Long-term potentiation
MAP 2: Microtubule-associated protein 2
mTOR: Mammalian target of rapamycin
PSC: Pluripotent stem cell
PSD: Postsynaptic density
RBP: RNA-binding protein
RNAseq: RNA sequencing
SFARI: Simons Foundation Autism Research Initiative
smFISH: Single-molecule fluorescence in situ hybridization
TSC: Tuberous sclerosis complex

## References

[1] Baio J, Wiggins L, Christensen DL (2018). Prevalence of autism spectrum disorder among children aged 8 years — autism and developmental disabilities monitoring network, 11 sites, United States, 2014. MMWR Surveill Summ.

[2] Hulbert SW, Jiang YH (2016). Monogenic mouse models of autism spectrum disorders: common mechanisms and missing links. Neuroscience..

[3] Peca J, Feng G (2012). Cellular and synaptic network defects in autism. Curr Opin Neurobiol.

[4] Bourgeron T (2015). From the genetic architecture to synaptic plasticity in autism spectrum disorder. Nat Rev Neurosci.

[5] Bourne JN, Harris KM (2007). Do thin spines learn to be mushroom spines that remember?. Curr Opin Neurobiol.

[6] Ziv NE, Smith SJ (1996). Evidence for a role of dendritic filopodia in synaptogenesis and spine formation. Neuron..

[7] Hayashi Y, Majewska AK (2005). Dendritic spine geometry: functional implication and regulation. Neuron..

[8] Alvarez VA, Sabatini BL (2007). Anatomical and physiological plasticity of dendritic spines. Annu Rev Neurosci.

[9] Arellano JI, Benavides-Piccione R, Defelipe J, Yuste R (2007). Ultrastructure of dendritic spines: correlation between synaptic and spine morphologies. Front Neurosci.

[10] Fiala JC, Feinberg M, Popov V, Harris KM (1998). Synaptogenesis via dendritic filopodia in developing hippocampal area CA1. J Neurosci.

[11] Tønnesen J, Katona G, Rózsa B, Nägerl UV (2014). Spine neck plasticity regulates compartmentalization of synapses. Nat Neurosci.

[12] Vanderklish PW, Edelman GM (2002). Dendritic spines elongate after stimulation of group 1 metabotropic glutamate receptors in cultured hippocampal neurons. Proc Natl Acad Sci U S A.

[13] Geschwind DH, Levitt P (2007). Autism spectrum disorders: developmental disconnection syndromes. Curr Opin Neurobiol.

[14] Hutsler JJ, Zhang H (2010). Increased dendritic spine densities on cortical projection neurons in autism spectrum disorders. Brain Res.

[15] Irwin SA, Patel B, Idupulapati M, Harris JB, Crisostomo RA, Larsen BP, Kooy F, Willems PJ, Cras P, Kozlowski PB, Swain RA, Weiler IJ, Greenough WT (2001). Abnormal dendritic spine characteristics in the temporal and visual cortices of patients with fragile-X syndrome: a quantitative examination. Am J Med Genet.

[16] Cruz-Martín A, Crespo M, Portera-Cailliau C (2010). Delayed stabilization of dendritic spines in fragile X mice. J Neurosci.

[17] Phillips M, Pozzo-Miller L (2015). Dendritic spine dysgenesis in autism related disorders. Neurosci Lett.

[18] Tavazoie SF, Alvarez VA, Ridenour DA, Kwiatkowski DJ, Sabatini BL (2005). Regulation of neuronal morphology and function by the tumor suppressors Tsc1 and Tsc2. Nat Neurosci.

[19] Yasuda S, Sugiura H, Katsurabayashi S, Shimada T, Tanaka H, Takasaki K (2014). Activation of Rheb, but not of mTORC1, impairs spine synapse morphogenesis in tuberous sclerosis complex. Sci Rep.

[20] Tang G, Gudsnuk K, Kuo S-H, Cotrin ML, Rosoklija AG, Sosunov A (2014). Loss of mTOR-dependent macroautophagy causes autistic-like synaptic pruning deficits. Neuron..

[21] Krepischi AC, Rosenberg C, Costa SS, Crolla JA, Huang S, Vianna-Morgante AM (2010). A novel de novo microdeletion spanning the SYNGAP1 gene on the short arm of chromosome 6 associated with mental retardation. Am J Med Genet A.

[22] Hamdan FF, Daoud H, Piton A, Gauthier J, Dobrzeniecka S, Krebs MO, Joober R, Lacaille JC, Nadeau A, Milunsky JM, Wang Z, Carmant L, Mottron L, Beauchamp MH, Rouleau GA, Michaud JL (2011). De novo SYNGAP1 mutations in nonsyndromic intellectual disability and autism. Biol Psychiatry.

[23] Srivastava DP, Jones KA, Woolfrey KM, Burgdorf J, Russell TA, Kalmbach A, Lee H, Yang C, Bradberry MM, Wokosin D, Moskal JR, Casanova MF, Waters J, Penzes P (2012). Social, communication, and cortical structural impairments in Epac2-deficient mice. J Neurosci.

[24] Steward O, Levy WB (1982). Preferential localization of polyribosomes under the base of dendritic spines in granule cells of the dentate gyrus. J Neurosci.

[25] Rao A, Steward O (1991). Evidence that protein constituents of postsynaptic membrane specializations are locally synthesized: analysis of proteins synthesized within synaptosomes. J Neurosci.

[26] Job C, Eberwine J (2001). Localization and translation of mRNA in dendrites and axons. Nat Rev Neurosci.

[27] Kleiman R, Banker G, Steward O (1990). Differential subcellular localization of particular mRNAs in hippocampal neurons in culture. Neuron..

[28] Hirokawa N, Takemura R (2005). Molecular motors and mechanisms of directional transport in neurons. Nat Rev Neurosci.

[29] Knowles RB, Sabry JH, Martone ME, Deerinck TJ, Ellisman MH, Bassell GJ, Kosik KS (1996). Translocation of RNA granules in living neurons. J Neurosci.

[30] Rook MS, Lu M, Kosik KS (2000). CaMKIIalpha 3′ untranslated region-directed mRNA translocation in living neurons: visualization by GFP linkage. J Neurosci.

[31] Kindler S, Kreienkamp HJ (2012). Dendritic mRNA targeting and translation. Adv Exp Med Biol.

[32] Zhao J, Fok AHK, Fan R, Kwan PY, Chan HL, Lo LH, Chan YS, Yung WH, Huang J, Lai CSW, Lai KO (2020). Specific depletion of the motor protein KIF5B leads to deficits in dendritic transport, synaptic plasticity and memory. eLife.

[33] Hüttelmaier S, Zenklusen D, Lederer M, Dictenberg J, Lorenz M, Meng X, Bassell GJ, Condeelis J, Singer RH (2005). Spatial regulation of beta-actin translation by Src-dependent phosphorylation of ZBP1. Nature.

[34] Tanaka J, Horiike Y, Matsuzaki M, Miyazaki T, Ellis-Davies GC, Kasai H (2008). Protein synthesis and neurotrophin-dependent structural plasticity of single dendritic spines. Science..

[35] Messaoudi E, Kanhema T, Soule J, Tiron A, Dagyte G, da Silva B, Bramham CR (2007). Sustained Arc/Arg3.1 synthesis controls longterm potentiation consolidation through regulation of local actin polymerization in the dentate gyrus in vivo. J Neurosci.

[36] Mayford M, Baranes D, Podsypanina K, Kandel ER (1996). The 3′-untranslated region of CaMKII alpha is a cis-acting signal for the localization and translation of mRNA in dendrites. Proc Natl Acad Sci U S A.

[37] Tongiorgi E, Righi M, Cattaneo A (1997). Activity-dependent dendritic targeting of BDNF and TrkB mRNAs in hippocampal neurons. J Neurosci.

[38] An JJ, Gharami K, Liao GY, Woo NH, Lau AG, Vanevski F, Torre ER, Jones KR, Feng Y, Lu B, Xu B (2008). Distinct role of long 3′ UTR BDNF mRNA in spine morphology and synaptic plasticity in hippocampal neurons. Cell..

[39] Miller S, Yasuda M, Coats JK, Jones Y, Martone ME, Mayford M (2002). Disruption of dendritic translation of CaMKIIalpha impairs stabilization of synaptic plasticity and memory consolidation. Neuron..

[40] Orefice LL, Waterhouse EG, Partridge JG, Lalchandani RR, Vicini S, Xu B (2013). Distinct roles for somatically and dendritically synthesized brain-derived neurotrophic factor in morphogenesis of dendritic spines. J Neurosci.

[41] Chen ZY, Jing D, Bath KG, Ieraci A, Khan T, Siao CJ, Herrera DG, Toth M, Yang C, McEwen BS, Hempstead BL, Lee FS (2006). Genetic variant BDNF (Val66Met) polymorphism alters anxiety-related behavior. Science..

[42] Chiaruttini C, Vicario A, Li Z, Baj G, Braiuca P, Wu Y, Lee FS, Gardossi L, Baraban JM, Tongiorgi E (2009). Dendritic trafficking of BDNF mRNA is mediated by translin and blocked by the G196A (Val66Met) mutation. Proc Natl Acad Sci U S A.

[43] Cajigas IJ, Tushev G, Will TJ, Tom Dieck S, Fuerst N, Schuman EM (2012). The local transcriptome in the synaptic neuropil revealed by deep sequencing and high-resolution imaging. Neuron.

[44] Ainsley JA, Drane L, Jacobs J, Kittelberger KA, Reijmers LG (2014). Functionally diverse dendritic mRNAs rapidly associate with ribosomes following a novel experience. Nat Commun.

[45] Middleton SA, Eberwine J, Kim J (2019). Comprehensive catalog of dendritically localized mRNA isoforms from sub-cellular sequencing of single mouse neurons. BMC Biol.

[46] Liang Z, Zhan Y, Shen Y, Wong CC, Yates JR, Plattner F, Lai KO, Ip NY (2016). The pseudokinase CaMKv is required for the activity-dependent maintenance of dendritic spines. Nat Commun.

[47] Lin L, Lo LH, Lyu Q, Lai KO (2017). Determination of dendritic spine morphology by the striatin scaffold protein STRN4 through interaction with the phosphatase PP2A. J Biol Chem.

[48] Lin L, Lyu Q, Kwan PY, Zhao J, Fan R, Chai A, Lai CSW, Chan YS, Shen X, Lai KO (2020). The epilepsy and intellectual disability-associated protein TBC1D24 regulates the maintenance of excitatory synapses and animal behaviors. PLoS Genet.

[49] Sala C, Vicidomini C, Bigi I, Mossa A, Verpelli C (2015). Shank synaptic scaffold proteins: keys to understanding the pathogenesis of autism and other synaptic disorders. J Neurochem.

[50] Durand CM, Perroy J, Loll F, Perrais D, Fagni L, Bourgeron T, Montcouquiol M, Sans N (2011). SHANK3 mutations identified in autism lead to modification of dendritic spine morphology via an actin-dependent mechanism. Mol Psychiatry.

[51] Napoli I, Mercaldo V, Boyl PP, Eleuteri B, Zalfa F, De Rubeis S, Di Marino D, Mohr E, Massimi M, Falconi M, Witke W, Costa-Mattioli M, Sonenberg N, Achsel T, Bagni C (2008). The fragile X syndrome protein represses activity-dependent translation through CYFIP1, a new 4E-BP. Cell..

[52] Davenport EC, Szulc BR, Drew J, Taylor J, Morgan T, Higgs NF, López-Doménech G, Kittler JT (2019). Autism and schizophrenia-associated CYFIP1 regulates the balance of synaptic excitation and inhibition. Cell Rep.

[53] Kelleher RJ, Bear MF (2008). The autistic neuron: troubled translation?. Cell..

[54] Suzuki AM, Griesi-Oliveira K, de Oliveira Freitas Machado C, Vadasz E, Zachi EC, Passos-Bueno MR, Sertie AL (2015). Altered mTORC1 signaling in multipotent stem cells from nearly 25% of patients with nonsyndromic autism spectrum disorders. Mol Psychiatry.

[55] Dölen G, Osterweil E, Rao BS, Smith GB, Auerbach BD, Chattarji S, Bear MF (2007). Correction of fragile X syndrome in mice. Neuron..

[56] Gkogkas CG, Khoutorsky A, Ran I, Rampakakis E, Nevarko T, Weatherill DB, Vasuta C, Yee S, Truitt M, Dallaire P, Major F, Lasko P, Ruggero D, Nader K, Lacaille JC, Sonenberg N (2013). Autism-related deficits via dysregulated eIF4E-dependent translational control. Nature..

[57] Bagni C, Tassone F, Neri G, Hagerman R (2012). Fragile X syndrome: causes, diagnosis, mechanisms, and therapeutics. J Clin Invest.

[58] Kashima R, Roy S, Ascano M, Martinez-Cerdeno V, Ariza-Torres J, Kim S, Louie J, Lu Y, Leyton P, Bloch KD, Kornberg TB, Hagerman PJ, Hagerman R, Lagna G, Hata A (2016). Augmented noncanonical BMP type II receptor signaling mediates the synaptic abnormality of fragile X syndrome. Sci Signal.

[59] Basu SN, Kollu R, Banerjee-Basu S (2009). AutDB: a gene reference resource for autism research. Nucleic Acids Res.

[60] Darnell JC, Van Driesche SJ, Zhang C, Hung KY, Mele A, Fraser CE, Stone EF, Chen C, Fak JJ, Chi SW, Licatalosi DD, Richter JD, Darnell RB (2011). FMRP stalls ribosomal translocation on mRNAs linked to synaptic function and autism. Cell..

[61] Bagni C, Greenough WT (2005). From mRNP trafficking to spine dysmorphogenesis: the roots of fragile X syndrome. Nat Rev Neurosci.

[62] Santini E, Huynh TN, MacAskill AF, Carter AG, Pierre P, Ruggero D, Kaphzan H, Klann E (2013). Exaggerated translation causes synaptic and behavioural aberrations associated with autism. Nature.

[63] Troca-Marín JA, Alves-Sampaio A, Montesinos ML (2012). Deregulated mTOR-mediated translation in intellectual disability. Prog Neurobiol.

[64] Costa-Mattioli M, Monteggia LM (2013). mTOR complexes in neurodevelopmental and neuropsychiatric disorders. Nat Neurosci.

[65] Parras A, Anta H, Santos-Galindo M, Swarup V, Elorza A, Nieto-González JL, Picó S, Hernández IH, Díaz-Hernández JI, Belloc E, Rodolosse A, Parikshak NN, Peñagarikano O, Fernández-Chacón R, Irimia M, Navarro P, Geschwind DH, Méndez R, Lucas JJ (2018). Autism-like phenotype and risk gene mRNA deadenylation by CPEB4 mis-splicing. Nature..

[66] Weyn-Vanhentenryck SM, Mele A, Yan Q, Sun S, Farny N, Zhang Z, Xue C, Herre M, Silver PA, Zhang MQ, Krainer AR, Darnell RB, Zhang C (2014). HITS-CLIP and integrative modeling define the Rbfox splicing-regulatory network linked to brain development and autism. Cell Rep.

[67] Lee JA, Damianov A, Lin CH, Fontes M, Parikshak NN, Anderson ES, Geschwind DH, Black DL, Martin KC (2016). Cytoplasmic Rbfox1 regulates the expression of synaptic and autism-related genes. Neuron..

[68] Tran SS, Jun HI, Bahn JH, Azghadi A, Ramaswami G, Van Nostrand EL, Nguyen TB, Hsiao YE, Lee C, Pratt GA, Martínez-Cerdeño V, Hagerman RJ, Yeo GW, Geschwind DH, Xiao X (2019). Widespread RNA editing dysregulation in brains from autistic individuals. Nat Neurosci.

[69] La Via L, Bonini D, Russo I, Orlandi C, Barlati S, Barbon A (2013). Modulation of dendritic AMPA receptor mRNA trafficking by RNA splicing and editing. Nucleic Acids Res.

[70] Tushev G, Glock C, Heumüller M, Biever A, Jovanovic M, Schuman EM (2018). Alternative 3′ UTRs modify the localization, regulatory potential, stability, and plasticity of mRNAs in neuronal compartments. Neuron..

[71] Takahashi K, Yamanaka S (2006). Induction of pluripotent stem cells from mouse embryonic and adult fibroblast cultures by defined factors. Cell..

[72] D'Aiuto L, Zhi Y, Kumar Das D, Wilcox MR, Johnson JW, McClain L, MacDonald ML, Di Maio R, Schurdak ME, Piazza P, Viggiano L, Sweet R, Kinchington PR, Bhattacharjee AG, Yolken R, Nimgaonkar VL (2014). Large-scale generation of human iPSC-derived neural stem cells/early neural progenitor cells and their neuronal differentiation. Organogenesis..

[73] Bigler RL, Kamande JW, Dumitru R, Niedringhaus M, Taylor AM (2017). Messenger RNAs localized to distal projections of human stem cell derived neurons. Sci Rep.

[74] Zappulo A, van den Bruck D, Ciolli Mattioli C, Franke V, Imami K, McShane E, Moreno-Estelles M, Calviello L, Filipchyk A, Peguero-Sanchez E, Müller T, Woehler A, Birchmeier C, Merino E, Rajewsky N, Ohler U, Mazzoni EO, Selbach M, Akalin A, Chekulaeva M (2017). RNA localization is a key determinant of neurite-enriched proteome. Nat Commun.

[75] Kathuria A, Nowosiad P, Jagasia R, Aigner S, Taylor RD, Andreae LC, Gatford NJF, Lucchesi W, Srivastava DP, Price J (2018). Stem cell-derived neurons from autistic individuals with SHANK3 mutation show morphogenetic abnormalities during early development. Stem cell-derived neurons from autistic individuals with SHANK3 mutation show morphogenetic abnormalities during early development. Mol Psychiatry.

[76] Gouder L, Vitrac A, Goubran-Botros H, Danckaert A, Tinevez JY, André-Leroux G, Atanasova E, Lemière N, Biton A, Leblond CS, Poulet A, Boland A, Deleuze JF, Benchoua A, Delorme R, Bourgeron T, Cloëz-Tayarani I (2019). Altered spinogenesis in iPSC-derived cortical neurons from patients with autism carrying de novo SHANK3 mutations. Sci Rep.

[77] Taylor SE, Taylor RD, Price J, Andreae LC (2018). Single-molecule fluorescence in-situ hybridization reveals that human SHANK3 mRNA expression varies during development and in autism-associated SHANK3 heterozygosity. Stem Cell Res Ther.

[78] Böckers TM, Segger-Junius M, Iglauer P, Bockmann J, Gundelfinger ED, Kreutz MR, Richter D, Kindler S, Kreienkamp HJ (2004). Mol Cell Neurosci.

[79] Li Y, Wang H, Muffat J, Cheng AW, Orlando DA, Lovén J, Kwok SM, Feldman DA, Bateup HS, Gao Q, Hockemeyer D, Mitalipova M, Lewis CA, Vander Heiden MG, Sur M, Young RA, Jaenisch R (2013). Global transcriptional and translational repression in human-embryonic-stem-cell-derived Rett syndrome neurons. Cell Stem Cell.

[80] Chekulaeva M, Landthaler M (2016). Eyes on translation. Mol Cell.

[81] Costa V, Aigner S, Vukcevic M, Sauter E, Behr K, Ebeling M, Dunkley T, Friedlein A, Zoffmann S, Meyer CA, Knoflach F, Lugert S, Patsch C, Fjeldskaar F, Chicha-Gaudimier L, Kiialainen A, Piraino P, Bedoucha M, Graf M, Jessberger S, Ghosh A, Bischofberger J, Jagasia R (2016). mTORC1 Inhibition corrects neurodevelopmental and synaptic alterations in a human stem cell model of tuberous sclerosis. Cell Rep.

[82] Sundberg M, Tochitsky I, Buchholz DE, Winden K, Kujala V, Kapur K, Cataltepe D, Turner D, Han MJ, Woolf CJ, Hatten ME, Sahin M (2018). Purkinje cells derived from TSC patients display hypoexcitability and synaptic deficits associated with reduced FMRP levels and reversed by rapamycin. Mol Psychiatry.

[83] Winden KD, Sundberg M, Yang C, Wafa SMA, Dwyer S, Chen PF, Buttermore ED, Sahin M (2019). Biallelic mutations in TSC2 lead to abnormalities associated with cortical tubers in human iPSC-derived neurons. J Neurosci.

[84] Banerjee A, Ifrim MF, Valdez AN, Raj N, Bassell GJ (2018). Aberrant RNA translation in fragile X syndrome: from FMRP mechanisms to emerging therapeutic strategies. Brain Res.

[85] Sheridan SD, Theriault KM, Reis SA, Zhou F, Madison JM, Daheron L, Loring JF, Haggarty SJ (2011). Epigenetic characterization of the FMR1 gene and aberrant neurodevelopment in human induced pluripotent stem cell models of fragile X syndrome. PLoS One.

[86] Doers ME, Musser MT, Nichol R, Berndt ER, Baker M, Gomez TM, Zhang SC, Abbeduto L, Bhattacharyya A (2014). iPSC-derived forebrain neurons from FXS individuals show defects in initial neurite outgrowth. Stem Cells Dev.

[87] Telias M, Kuznitsov-Yanovsky L, Segal M, Ben-Yosef D (2015). Functional deficiencies in fragile X neurons derived from human embryonic stem cells. J Neurosci.

[88] Liu J, Koscielska KA, Cao Z, Hulsizer S, Grace N, Mitchell G, Nacey C, Githinji J, McGee J, Garcia-Arocena D, Hagerman RJ, Nolta J, Pessah IN, Hagerman PJ (2012). Signaling defects in iPSC-derived fragile X premutation neurons. Hum Mol Genet.

[89] Abekhoukh S, Bardoni B (2014). CYFIP family proteins between autism and intellectual disability: links with fragile X syndrome. Font Cell Neurosci.

[90] Das DK, Tapias V, D'Aiuto L, Chowdari KV, Francis L, Zhi Y, Ghosh BA, Surti U, Tischfield J, Sheldon M, Moore JC, Fish K, Nimgaonkar V (2015). Genetic and morphological features of human iPSC-derived neurons with chromosome 15q11.2 (BP1-BP2) deletions. Mol Neuropsychiatry.

[91] Dimitrion P, Zhi Y, Clayton D, Apodaca GL, Wilcox MR, Johnson JW, Nimgaonkar V, D'Aiuto L (2017). Low-density neuronal cultures from human induced pluripotent stem cells. Mol Neuropsychiatry.

[92] Hiragi T, Andoh M, Araki T, Shirakawa T, Ono T, Koyama R, Ikegaya Y (2017). Differentiation of human induced pluripotent stem cell (hiPSC)-derived neurons in mouse hippocampal slice cultures. Front Cell Neurosci.

[93] Qian X, Jacob F, Song MM, Nguyen HN, Song H, Ming GL (2018). Generation of human brain region-specific organoids using a miniaturized spinning bioreactor. Nat Protoc.

[94] Quadrato G, Nguyen T, Macosko EZ, Sherwood JL, Min Yang S, Berger DR, Maria N, Scholvin J, Goldman M, Kinney JP, Boyden ES, Lichtman JW, Williams ZM, McCarroll SA (2017). Arlotta P1. Cell diversity and network dynamics in photosensitive human brain organoids. Nature.

[95] Penna E, Cerciello A, Chambery A, Russo R, Cernilogar FM, Pedone EM, Perrone-Capano C, Cappello S, Di Giaimo R, Crispino M (2019). Cystatin B Involvement in synapse physiology of rodent brains and human cerebral organoids. Front Mol Neurosci.

[96] Merkurjev D, Hong WT, Iida K, Oomoto I, Goldie BJ, Yamaguti H, Ohara T, Kawaguchi SY, Hirano T, Martin KC, Pellegrini M, Wang DO (2018). Synaptic N6-methyladenosine (m6A) epitranscriptome reveals functional partitioning of localized transcripts. Nat Neurosci.

[97] Hafner Anne-Sophie, Donlin-Asp Paul G., Leitch Beulah, Herzog Etienne, Schuman Erin M. (2019). Local protein synthesis is a ubiquitous feature of neuronal pre- and postsynaptic compartments. Science.

